# Fine-scale maps of malaria incidence to inform risk stratification in Laos

**DOI:** 10.1186/s12936-024-05007-9

**Published:** 2024-06-25

**Authors:** Su Yun Kang, Punam Amratia, Julia Dunn, Phoutnalong Vilay, Mark Connell, Tasmin Symons, Susan Rumisha, Song Zhang, Abigail Ward, Odai Sichanthongthip, Virasack Banouvong, Mathew Shortus, Rita Reyburn, Phonephet Butphomvihane, Vilaisak Phiphakavong, Mary Hahm, Vilayphone Phongchantha, Boualam Khamlome, Keobouphaphone Chindavongsa, Chitsavang Chanthavisouk, Daniel J. Weiss, Peter W. Gething, Ewan Cameron

**Affiliations:** 1 Telethon Kids Institute, Perth Children’s Hospital, Perth, Australia; 2 Ifakara Health Institute, Dar es Salaam, Tanzania; 3 Clinton Health Access Initiative, Boston, USA; 4 Centre of Malariology, Parasitology and Entomology, Vientiane, Lao PDR; 5 Curtin University, Perth, Australia; 6 World Health Organization, Vientiane, Lao PDR

## Abstract

**Background:**

Malaria risk maps are crucial for controlling and eliminating malaria by identifying areas of varying transmission risk. In the Greater Mekong Subregion, these maps guide interventions and resource allocation. This article focuses on analysing changes in malaria transmission and developing fine-scale risk maps using five years of routine surveillance data in Laos (2017–2021). The study employed data from 1160 geolocated health facilities in Laos, along with high-resolution environmental data.

**Methods:**

A Bayesian geostatistical framework incorporating population data and treatment-seeking propensity was developed. The models incorporated static and dynamic factors and accounted for spatial heterogeneity.

**Results:**

Results showed a significant decline in malaria cases in Laos over the five-year period and a shift in transmission patterns. While the north became malaria-free, the south experienced ongoing transmission with sporadic outbreaks.

**Conclusion:**

The risk maps provided insights into changing transmission patterns and supported risk stratification. These risk maps are valuable tools for malaria control in Laos, aiding resource allocation, identifying intervention gaps, and raising public awareness. The study enhances understanding of malaria transmission dynamics and facilitates evidence-based decision-making for targeted interventions in high-risk areas.

**Supplementary Information:**

The online version contains supplementary material available at 10.1186/s12936-024-05007-9.

## Background

Effective malaria control measures and increased funding for malaria programmes have led to a substantial decrease in malaria cases and deaths in the Greater Mekong Subregion (GMS) in recent years [1]. However, significant challenges continue to persist in the GMS, namely, the emergence and spread of anti-malarial drug-resistance for *Plasmodium falciparum* [2, 3], the presence of insecticide resistance among mosquito populations [4], the fluidity of cross-border movement between high transmission intensity regions and low transmission regions, and difficulty in reaching vulnerable populations in remote areas [5, 6].

Despite these hindrances, the Lao People’s Democratic Republic, commonly known as Laos, has made considerable efforts to combat malaria. These efforts involve enhancing access to diagnosis and treatment, implementing targeted vector control interventions (particularly for vulnerable populations such as forest-goers), deploying community-based malaria control programmes including insecticide-treated bed nets distribution, and cross-border collaboration [7]. However, formidable obstacles persist, such as remote transmission areas and the emergence of drug-resistant strains. Therefore, sustained collaboration and increased investment are imperative for achieving malaria elimination goals in Laos and the GMS.

Between 2010 and 2019, Laos witnessed an impressive 94% decrease in malaria cases and a 99% decrease in malaria-related deaths [8]. In 2021, only 35% of reported cases were *P. falciparum* infections, compared to 99% in 2009. Approximately 85% of all reported malaria cases in Laos were reported in three provinces: Xekong, Attapeu, and Salavan [9]. Under the guidance of the World Health Organization’s (WHO) Global Technical Strategy for malaria [10], Laos successfully implemented phase 1 of its National Strategic Plan to eliminate *P. falciparum* in northern and central provinces and reduce the annual incidence to less than 5 cases per 1000 population in southern provinces by 2020. By 2025, Laos aims to eliminate *P. falciparum* and *Plasmodium vivax* from all northern provinces and eliminate *P. falciparum* in the five southernmost provinces [7]. As Laos approaches elimination, it has been experiencing shifts in its malaria transmission. An increasing number of areas in the north have become malaria-free, with occasional focal outbreaks detected through routine surveillance systems, while the five southernmost provinces remain an area of stable transmission, characterized by continual geographical fluctuations [8]. These ever-changing shifts in the transmission profile have made it increasingly important to have a strong and enhanced surveillance system that can monitor the burden of the disease at the sub-national level.

Understanding the risk landscape of diseases is a critical part of any disease surveillance system and becomes even more important when striving for elimination. In malaria, risk is often described through the use of predicted maps, which are a graphical depiction of regions with high, moderate, or low risk of transmission. They are created by analysing data on malaria burden such as incidence or prevalence, vector distribution [4], environmental covariates, and other pertinent factors [11]. In the absence of truly complete data, geostatistical models offer the ability to predict the expected risk to unobserved space and provide a complete picture of transmission [12]. For example, fine-scale risk maps have been designed to support evidence-based risk stratification in Haiti, where prevalence is less than 1% [13]. In China, prior to certification, risk maps were used in identifying hotspots near the China-Myanmar border [14].

The maps from geostatistical approaches offer multiple benefits for malaria control and elimination programmes. Firstly, they enable efficient allocation of limited resources by helping to prioritize resources and target interventions in areas with high risk of malaria transmission, thus achieving maximum impact [15]. Secondly, they facilitate the identification of gaps in existing malaria control measures, allowing for the design of appropriate interventions to address these gaps. For example, in areas with high risk of malaria transmission but low coverage of insecticide-treated bed nets, health authorities can prioritize the distribution of bed nets [16]. Thirdly, malaria risk maps can be used to identify areas of persistent malaria that may need social behaviour change communications interventions, encouraging people to take preventive measures such as using bed nets, seeking early diagnosis and treatment, and avoiding mosquito bites [15].

A series of fine-scale maps was produced in collaboration with the Centre of Malariology, Parasitology and Entomology (CMPE), Clinton Health Access Initiative (CHAI) and WHO to support malaria risk stratification in Laos [17]. These maps were used to validate confirmed malaria case data from health facilities for their health facility catchment area. They also provided estimates of malaria cases when data from health facilities were deemed unreliable due to low reporting and/or testing rates. The study employed a novel Bayesian geostatistical framework to analyse routine surveillance malaria case data spanning from 2017 to 2021 in Laos. The current framework incorporated the population’s treatment-seeking behaviour, high-resolution environmental covariates, and health facility catchment population. The incidence rates were estimated at each health facility in the country, as well as at a spatial resolution of 1 km $\times$ 1 km for the entire country. Risk maps that covered a five-year period were presented, offering valuable insights into the spatio-temporal changes in malaria transmission and a detailed breakdown by species for the most recent three years, 2019 to 2021.

Through this comprehensive framework, the study enhanced understanding of malaria dynamics, informs risk stratification, and facilitates targeted interventions. The resulting fine-scale risk maps offered a valuable resource for malaria control and elimination programmes, aiding in resource allocation, intervention planning, and public health awareness campaigns in Laos.

## Methods

### Routine surveillance data

**Fig. 1:**
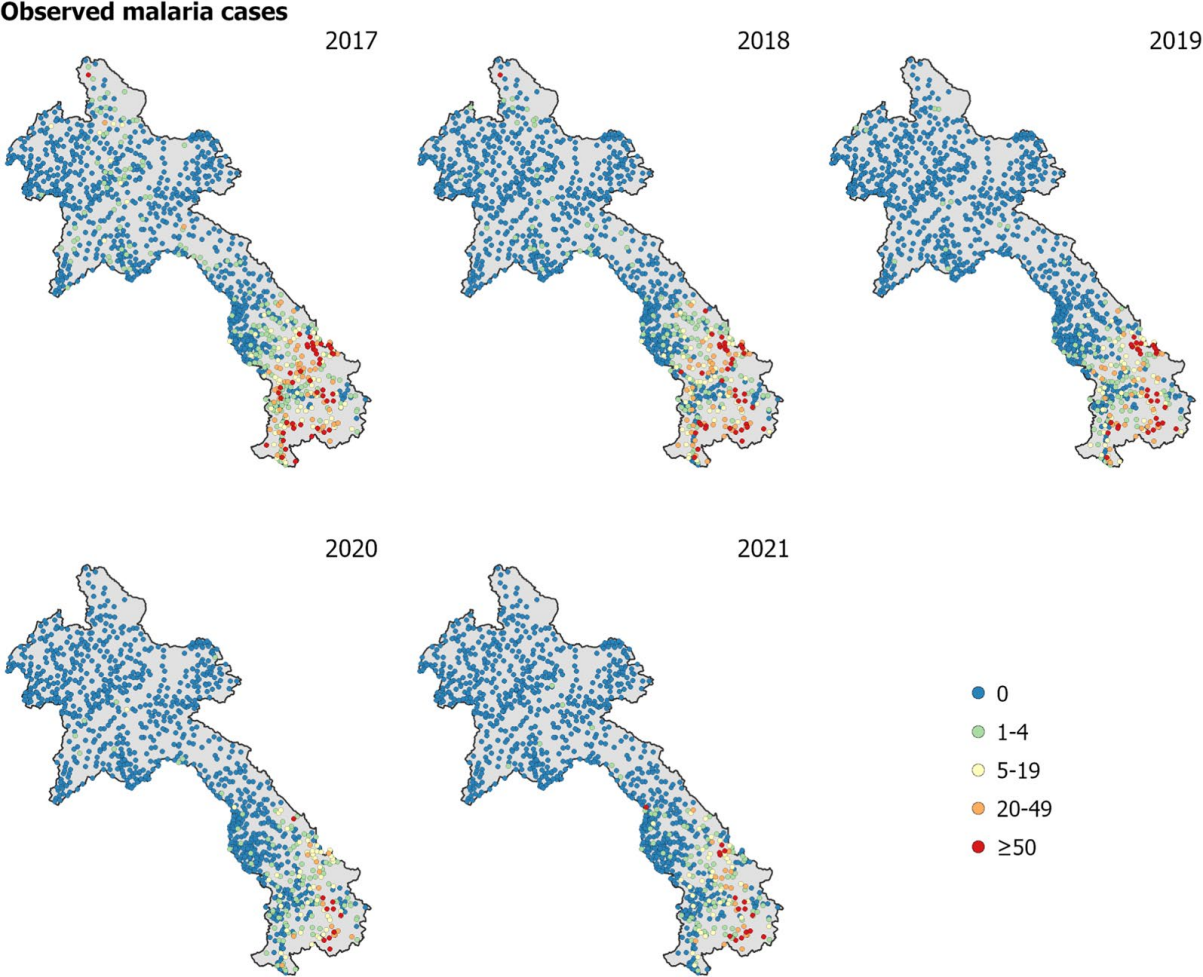
Observed malaria cases (*P. falciparum* and *P. vivax* combined) per year at health facilities across Laos. The observed malaria cases have considerably declined in Laos over the five years

The primary dataset comprised of annual malaria case counts from 2017 to 2021 for 1243 health facilities in Laos. As 83 health facilities did not have spatial coordinates, the analysis was performed on 1160 geolocated health facility points (health facilities with longitude and latitude). Figure 1 displays the observed malaria cases at health facilities throughout Laos from 2017 to 2021. The number of reported malaria cases had considerably declined in Laos over the five years, from approximately 9300 cases in 2017 to less than 4000 cases in both 2020 and 2021, with a further reduction to 2305 cases in 2022 [8]. The data from 2019 to 2021 included a breakdown by *P. falciparum* and *P. vivax* across health facilities in Laos [see Figure 1 in Supplementary Information (SI)].

### Treatment-seeking propensity

The continuous treatment-seeking propensity was estimated using a distance-decay-based model [18, 19] to any health facility. Here distance was defined as travel time using a road and terrain-informed friction surface from the Malaria Atlas Project [20]. The decay model was compared against survey data from the Lao PDR Social Indicator Survey II 2017 [21] (see Figure 2 in SI). According to the survey, the national treatment-seeking rate was 58%, with province-specific rates ranging from 28 to 77.8%. The minimum and maximum values of the decay curve were then calibrated to better match the national average.

### High resolution environmental covariates and model selection

The incidence model utilized a range of environmental covariates with a spatial resolution of 1 km $\times$ 1 km, that are known to influence and impact malaria outcomes. The selection of covariates was informed by a comprehensive review that identified robust associations with malaria (see [22]). The covariates are described in detail in Table 1. A suite of static and dynamic covariates were used.

**Table 1:** List of covariates

Static covariates	Description	Category
Access to cities [23]	Travel time distance to cities with population $> 50,000$	Urban/rural
AI [24]	Aridity Index	Vegetation
Distance to water [25]	GIS-derived surface that measures distance to permanent and semi-permanent water based on presence of lakes, wetlands, rivers and streams, and accounting for slope and precipitation	Hydrology
Elevation [26]	Elevation as measured by the shuttle radar topography mission (SRTM)	Topography
Night-time lights	Index that measures the presence of lights from towns, cities and other sites with persistent lighting	Urban/rural
PET [24]	Potential evapotranspiration	Hydrology
Population density	World population estimates, UN adjusted	Urban/rural
Slope [26]	GIS-derived surface calculated from SRTM elevation surface	Topography
TSI [27]	Temperature suitability index for P. falciparum	Temperature
Tree fraction	Percentage of forest cover change	Vegetation

Dynamic covariates	Description	Category
Rainfall [28]	Climate hazards group infrared precipitation with station data	Hydrology
EVI [29]	Enhanced vegetation index	Vegetation
LST Day [30]	Daytime land surface temperature	Temperature
LST Night [30]	Night-time land surface temperature	Temperature
TCB [31]	Tasselled cap brightness; measure of land reflectance	Vegetation
TCW [31]	Tasselled cap wetness; measure of land moisture	Hydrology

To evaluate the performance of the modelling approach, an extensive cross-validation analysis was conducted. This involved testing different model combinations, including a full model comprising both covariates and spatial random effects, a model with covariates only, and a model with spatial random effects only.

Additionally, the performance of two different parasite models were assessed using the data from 2019 to 2021 which included a breakdown by *P. falciparum* and *P. vivax*. The first model simultaneously fitted *P. falciparum* and *P. vivax*, while the second model fitted these two parasites separately. By comparing the performance of these different models, the most effective approach for predicting and understanding malaria transmission dynamics was determined.

Overall, the modelling approach used here provided valuable insights into the environmental factors that contributed to malaria transmission and helped identify high-risk areas for malaria. Furthermore, the comparison of different model combinations and parasite models aided in improving the accuracy and reliability of predictions and ultimately informed more effective malaria control strategies.

### Estimating catchment population

To calculate the appropriate incidence rates for the cases observed at health facilities, the specific catchment population for each health facility was estimated. Catchment population in this case was defined as the number of people likely to seek treatment at each facility. The population data were derived from High Resolution Settlement Layer (HRSL) representing population in Laos in 2018. District-level population estimates for $2019-2021$ were used to adjust for HRSL population estimates using raking. In this analysis, a modified gravity style model was used to estimate these catchment populations based on travel time to the health facilities, using the latest global maps of travel time to healthcare facilities [20].

For the catchment model, the probability that an individual in the *i*-th pixel seeked treatment at the *j*-th health facility, $\bar{p}(\text {pixel}_i \rightarrow \text {hf}_j)$, was modelled as the relative attractiveness of that facility, $p(\text {pixel}_i\rightarrow \text {hf}_j)$, normalized in relation to the relative attractiveness of all accessed facilities, $\sigma _i$, i.e.,

$$\begin{aligned} \bar{p}(\text {pixel}_i \rightarrow \text {hf}_j) = \frac{ p(\text {pixel}_i\rightarrow \text {hf}_j)}{\sum _{k \in \sigma _i} p(\text {pixel}_i\rightarrow \text {hf}_k)}. \end{aligned}$$

This relative attractiveness was modelled as the combination of an inherent decline proportional to the square of the travel time to that health facility, $tt(\text {pixel}_i \rightarrow \text {hf}_j)$, and a catchment weighting, $W_j$, intended to capture the variation in services available at each facility that would likely attract an individual seeking care, i.e.,

$$\begin{aligned} p(\text {pixel}_i \rightarrow \text {hf}_j) = \frac{W_j}{tt(\text {pixel}_i \rightarrow \text {hf}_j)^2}. \end{aligned}$$

The catchment weightings were treated as a model parameter to be inferred jointly with the clinical incidence rate during fitting, and a Bayesian prior of form $\text {log }W_j \sim \text {Normal}(0, 0.25^2)$ was used to regularise their variation. Combining this attractiveness model with the treatment-seeking and population surfaces—denoted here as $\text {TS}_i$ and $\text {population}_i$, respectively—allowed the catchment population of the *j*-th health facility to be defined as

$$\begin{aligned} \text {CP}_{j} = \sum \limits _{i=1}^{N_{\text {pixel}}} \text {TS}_i \times \text {population}_i \times \bar{p}(\text {pixel}_i \rightarrow \text {hf}_j). \end{aligned}$$

In the *i*-th pixel, residents’ access to facilities was limited to those within a 3-hour (180 min) travel time. It was assumed that individuals generally would not bypass numerous nearby health facilities to visit a more distant one. This assumption was reflected in the model, particularly in areas with sparse health facility distribution, where the accessible set, $\sigma _i$, included only the five nearest facilities. In contrast, in areas where several health facilities were within a 20-minute travel distance, $\sigma _i$ consisted of these facilities plus the next four nearest ones. Catchments were modelled as static and did not vary on a monthly nor yearly basis, mainly owing to lack of information on dynamic activities of health facilities and treatment-seeking rates.

### Incidence model

A Bayesian geostatistical framework was employed to represent the underlying incidence rate at each pixel, *i*, for each year, *y*, with the above catchment model connecting this latent risk surface, $I_{i,y}$, to the available case data.

For each year, the matrix of covariates, $\textbf{X}_y$, was formed by joining the collection of static covariates with the time-varying covariates matching that year. The linear predictor for the *i*-th pixel in year *y* was then formed by multiplication of the *i*-th row in $\textrm{X}_y$ against an annual slope vector, $\beta _y$. A fixed annual intercept term, $c_y$ and an annual spatial offset term, $f_{\textrm{offset},i,y}$, completed the formula for $I_{i,y}$ as

$$\begin{aligned} \text {log }I_{i,y} = c_y + (\textbf{X}_y)^\prime _i\beta _y + f_{\textrm{offset},i,y}. \end{aligned}$$

The annual spatial offset term was drawn from a spatial Gaussian random field as

$$\begin{aligned} f_{\textrm{offset},\cdot ,y} \sim \textrm{GP}(\text {range}_{\text {offset}}, \text {scale}_{\text {offset}}) \end{aligned}$$

with the hyper-parameters assigned hyper-priors, $\textrm{range}_{\text {offset}} \sim \textrm{Normal}(1,0.5^2)$ and $\textrm{scale}_{\text {offset}} \sim \textrm{Normal}(-1,0.5^2)$. A modest shrinkage penalty was placed on the annual slope vector via the prior choice, $\beta _y \sim \textrm{Normal}(0,1^2)$.

The expected cases at the *j*-th facility in year *y*, were computed by summation over all pixels of the product of the latent incidence surface with the catchment model; namely,

$$\begin{aligned} \mathrm {expected\ cases}_{j,y} = \sum _i I_{i,y} \times \text {TS}_i \times \text {population}_i \times \bar{p}(\text {pixel}_i \rightarrow \text {hf}_j). \end{aligned}$$

To allow for over-dispersion relative to the nominal Poisson sampling distribution for case data, the annual case totals observed at each facility were drawn from a Negative Binomial distribution parameterized as

$$\begin{aligned} \text {cases}_{i,y} \sim \text {NegBinom}\left( \text {mean} = \mathrm {expected\ cases}_{i,y}, \text {variance} = \text {mean}\times (1+\sigma ) \right) \end{aligned}$$

with $\sigma$ assigned a hyper-prior, $\log \sigma \sim \text {Normal}(-1, 1^2)$.

Model fitting was performed in the Template Model Builder (TMB) and Integrated Nested Laplace Approximation (INLA) packages [32, 33] for R using a Laplace approximation over the random field components and the (logarithm of) catchment attractiveness weights, with a Multivariate Normal approximation in the remaining hyper-parameters centred on the empirical Bayes estimator. Model uncertainty was derived from 300 samples drawn from a Laplace approximation of the posterior and quantified using the standard deviation of the posterior distribution, as well as threshold exceedance and non-exceedance probabilities.

Data for the years 2019 to 2021 provided information on species breakdown to *P. falciparum* and *P. vivax* cases for all ages. The methodology described above was applied independently to each species, generating incidence maps tailored to individual species and averaged by year. To explore overlapping risk profiles, these species-specific incidence maps were superimposed, enabling a comprehensive assessment of risk both for individual species and their collective impact. The model validation outcomes, tables of goodness-of-fit measures, and diagnostic figures were included in the Supplementary material. These resources were intended to aid readers in evaluating the effectiveness of the geospatial model.

## Results

**Fig. 2:**
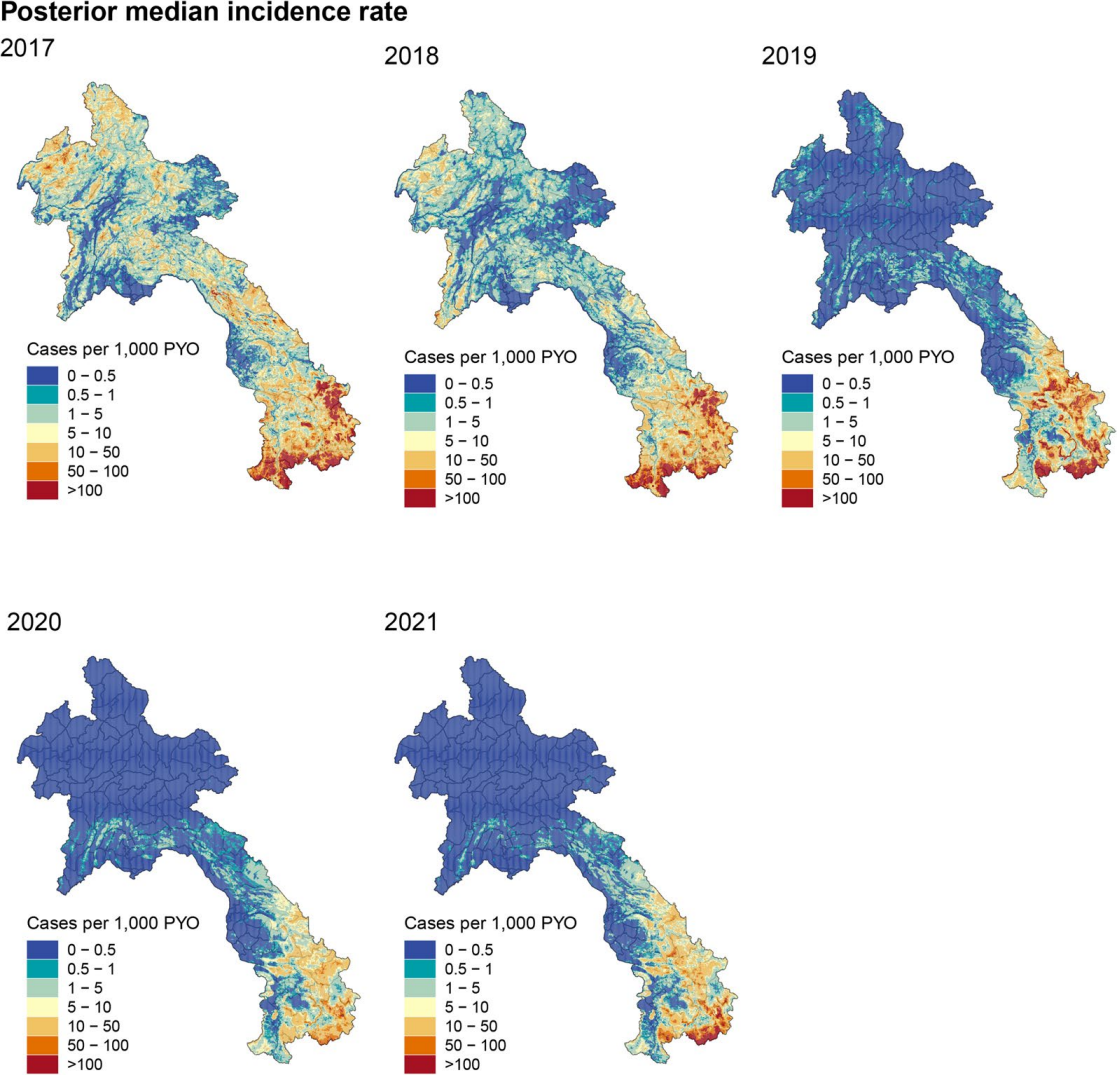
Fine-scale maps (1 km $\times$ 1 km) of the estimated median of annual malaria cases per 1000 person-year-observed in Laos for years 2017–2021 produced from the geospatial model. The maps highlight higher transmission rates in rural and remote areas compared to urban centers. Notably, the southern provinces of Sekong, Savannakhet, Salavan, Champasak, and Attapeu consistently emerge as high-risk areas for malaria transmission

By employing a Bayesian geostatistical framework fitted to the yearly routine surveillance data from 2017 to 2021 in Laos, valuable insights into the spatial distribution and trends of malaria incidence were obtained. Figure 2 displays the predicted malaria incidence in units of cases per 1000 person-year-observed (PYO) at a spatial resolution of 1 km $\times$ 1 km in Laos from 2017 to 2021. The maps vividly portray the geographical variations in malaria incidence, highlighting higher transmission rates in rural and remote areas compared to urban centres [34]. Notably, the southern provinces of Sekong, Savannakhet, Salavan, Champasak, and Attapeu are observed as persistently high-risk areas for malaria transmission. The maps clearly demonstrate a declining trend in malaria incidence rates over the course of the five-year study period. Particularly noteworthy is the significant reduction observed in the northern region, where the incidence dropped to less than 0.5 cases per 1000 PYO in 2020 and 2021, indicating substantial progress towards malaria elimination. In the context of the risk stratification exercise, it is noteworthy that the geospatial model demonstrates a robust fit to the observed data (see Figure 3 in SI).

**Fig. 3:**
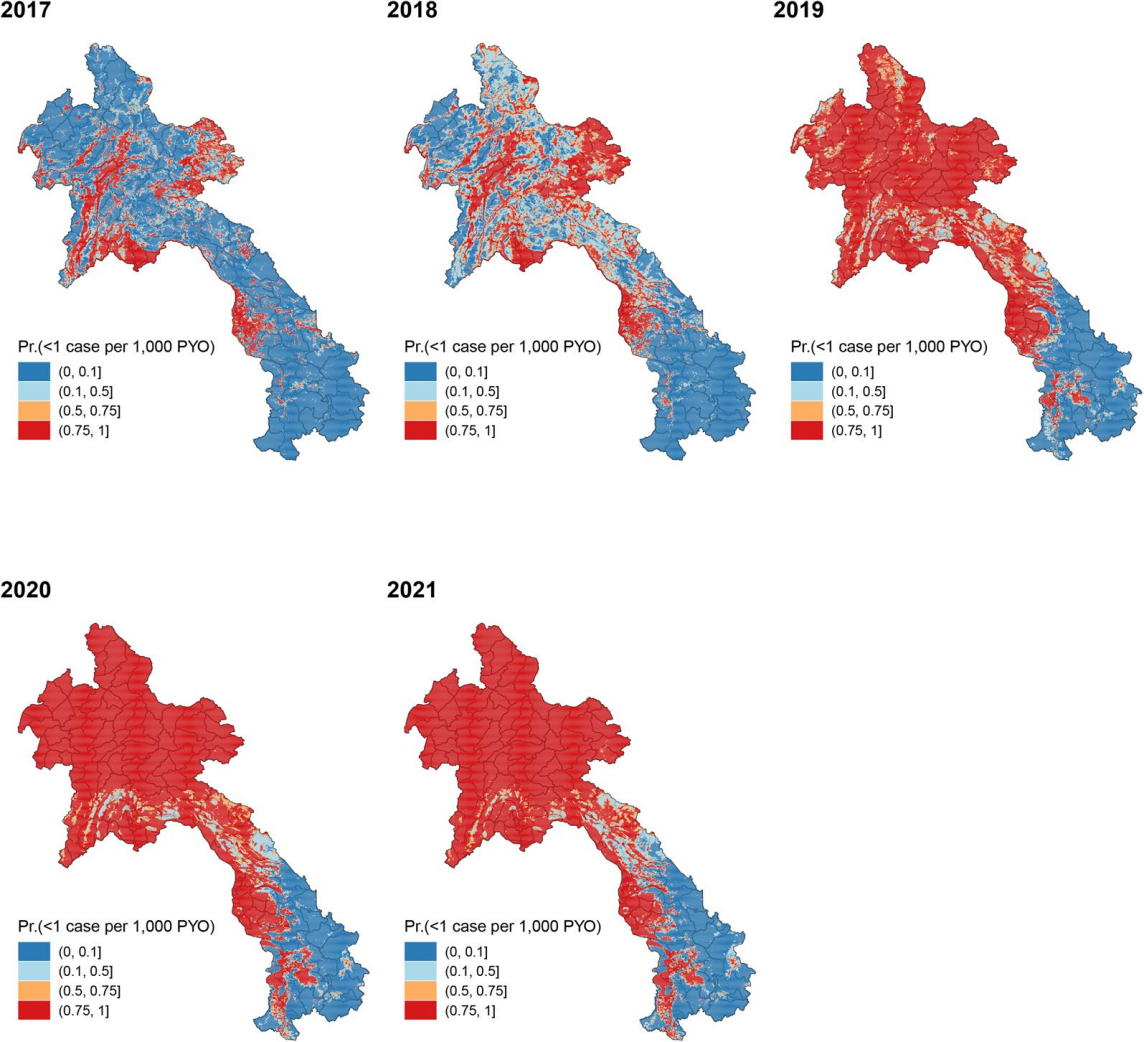
The posterior probability that the incidence cases of malaria in Laos do not exceed 1 case per 1000 person-year-observed in each pixel for years 2017–2021 based on the Bayesian geospatial model fit. The maps exhibit a growing level of certainty over the five years, signifying significant strides made towards malaria elimination in the majority of the country

Figure 3 presents the posterior probability that the malaria incidence in Laos exceeds the threshold of one case per 1000 PYO. This threshold is a pivotal benchmark according to the WHO’s directives for the elimination of malaria within the GMS [1]. The representation in Fig. 3 conveys the extent to which the observed incidence aligns with this elimination target, allowing for informed assessments of progress toward malaria elimination goals. Over the five-year period, the maps exhibit a growing level of certainty, signifying significant strides made towards malaria elimination in the majority of the country.

**Fig. 4:**
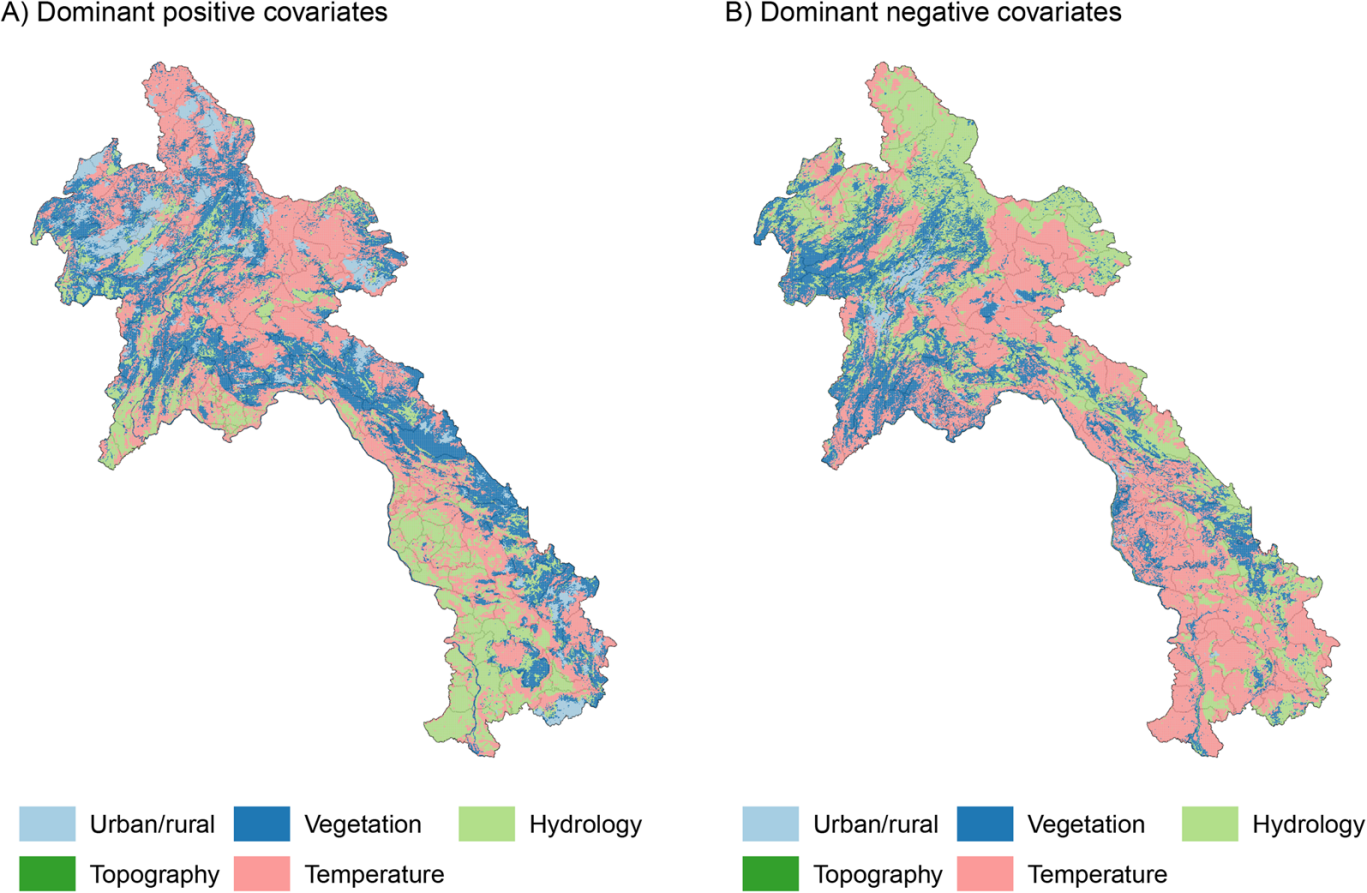
The dominant covariates in fine-scale prediction of the case incidence rate for Laos (averaged across 2017 to 2021). Each pixel’s color corresponds to the covariate exerting the **A** greatest positive impact (contributing to heightened local malaria risk estimates) and **B** greatest negative impact (leading to reduced local malaria risk estimates), aligned with the legend

The most influential covariates under the fitted model for the annual malaria incidence rate averaged across 2017 to 2021 are highlighted in Fig. 4, which shows the dominant positive and negative covariate in each pixel. To enhance interpretability, the 16 covariates outlined in Table 1 have been organized into distinct categories. These categories encompass Urban/Rural indicators (Access to cities, Night-time lights, Population density), Vegetation factors (AI, Tree fraction, EVI, TCB), Hydrology metrics (Distance to water, PET, Rainfall, TCW), Topographical attributes (Elevation, Slope), and Temperature variables (TSI, LST Day, LST Night). Of particular interest is the observation that factors related to urban/rural settings, hydrology, and vegetation seem to exhibit associations with an elevated risk of malaria transmission, particularly within the southern region of the country. Nonetheless, it is crucial to avoid interpreting these outcomes as indicative of causal significance. Figure 4 is presented solely to offer insights into the structure of the fitted regression model.

**Fig. 5:**
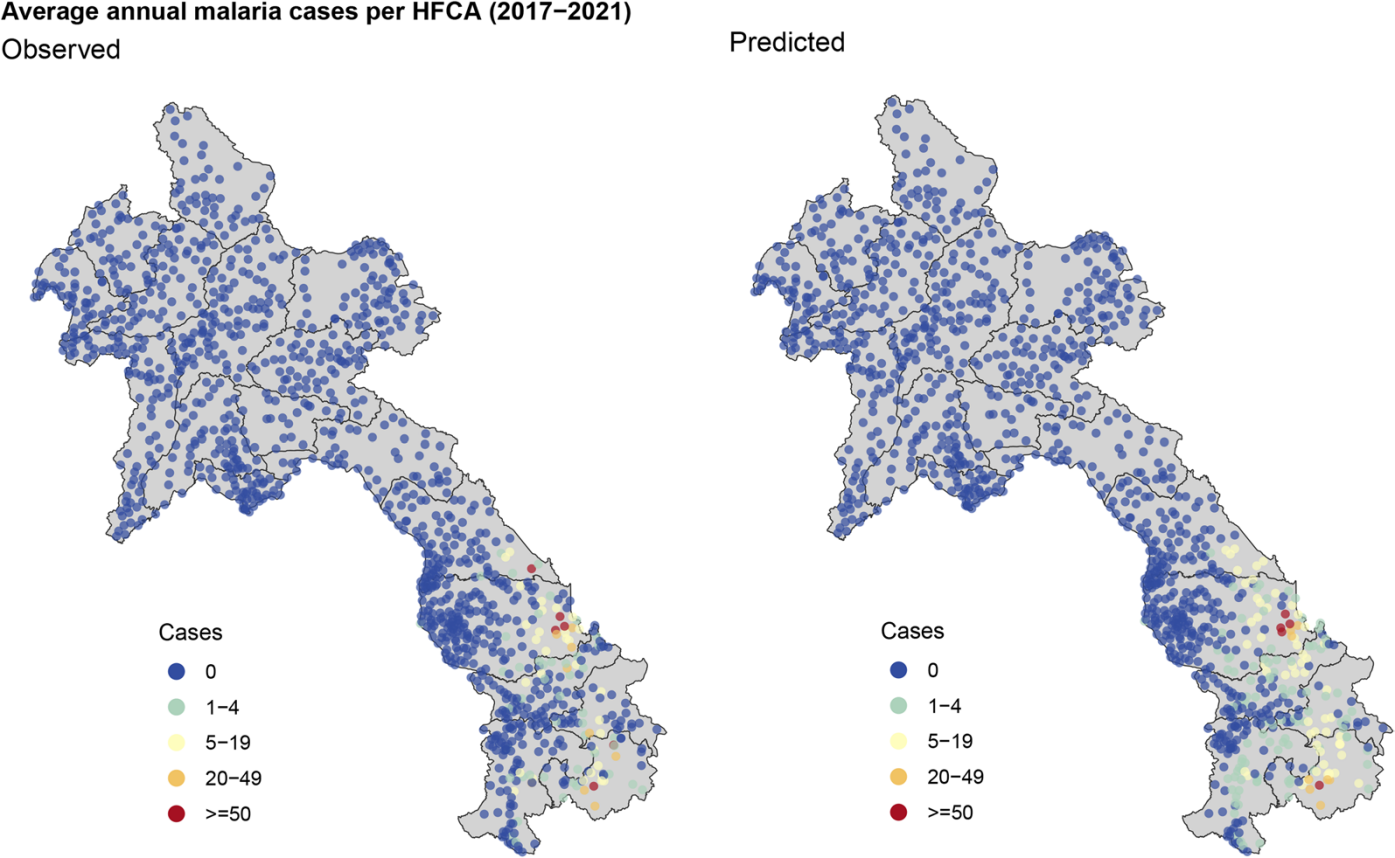
A comparison between the observed and predicted annual malaria cases within each health facility catchment area (HFCA), averaging across the years 2017 to 2021. The predicted map indicates the geospatial model’s predictive capabilities and its ability to estimate catchment populations effectively

Figure 5 illustrates a comparison between the observed and predicted annual malaria cases within each health facility catchment area, averaging across the years 2017 to 2021. With a strong resemblance evident in the two maps, the figure clearly demonstrates the geospatial model’s predictive capabilities and its ability to estimate catchment populations effectively (also see Figure 4 in SI).

**Fig. 6:**
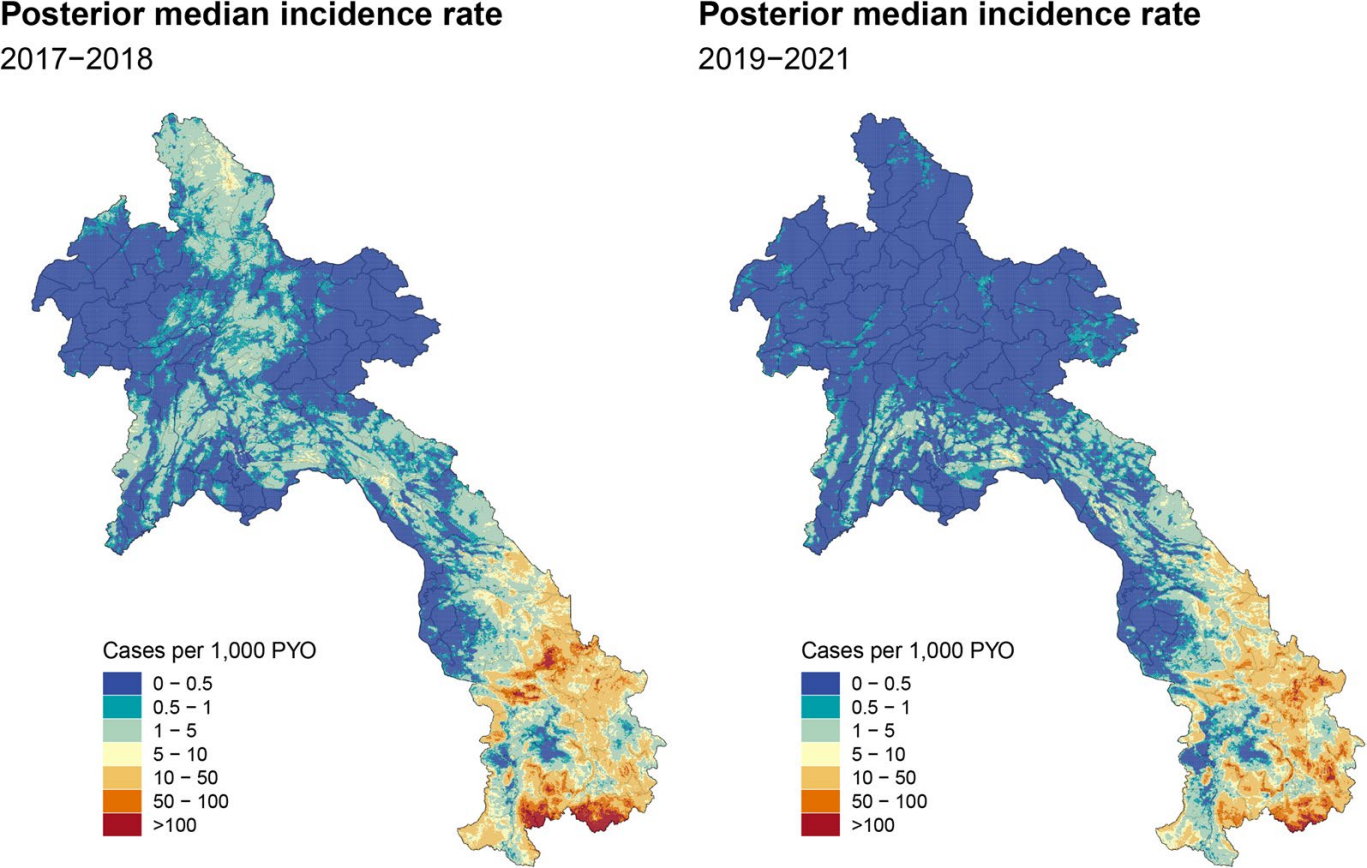
Fine-scale annual average risk maps for 2017–2018 and 2019–2021: The left panel shows the posterior median incidence rate for 2017–2018 while the right panel shows the same metric for 2019–2021. A substantial reduction in malaria transmission in the northern region of the country has been observed in the period of 2019–2021

Figure 6 displays the annual average of malaria incidence rates in Laos for two distinct periods: 2017–2018 and 2019–2021. The final model estimates were averaged across the two and three years, respectively, to provide an annual average estimate. These maps played a pivotal role in informing risk stratification efforts, specifically in 2019 and 2022, respectively [17]. Notably, the map representing the period of 2019–2021 demonstrates a substantial reduction in malaria transmission in the northern region of the country. Malaria incidence rates in this region predominantly fell below the threshold of 0.5 cases per 1000 PYO, highlighting the effectiveness of malaria control interventions implemented in these areas.

**Fig. 7:**
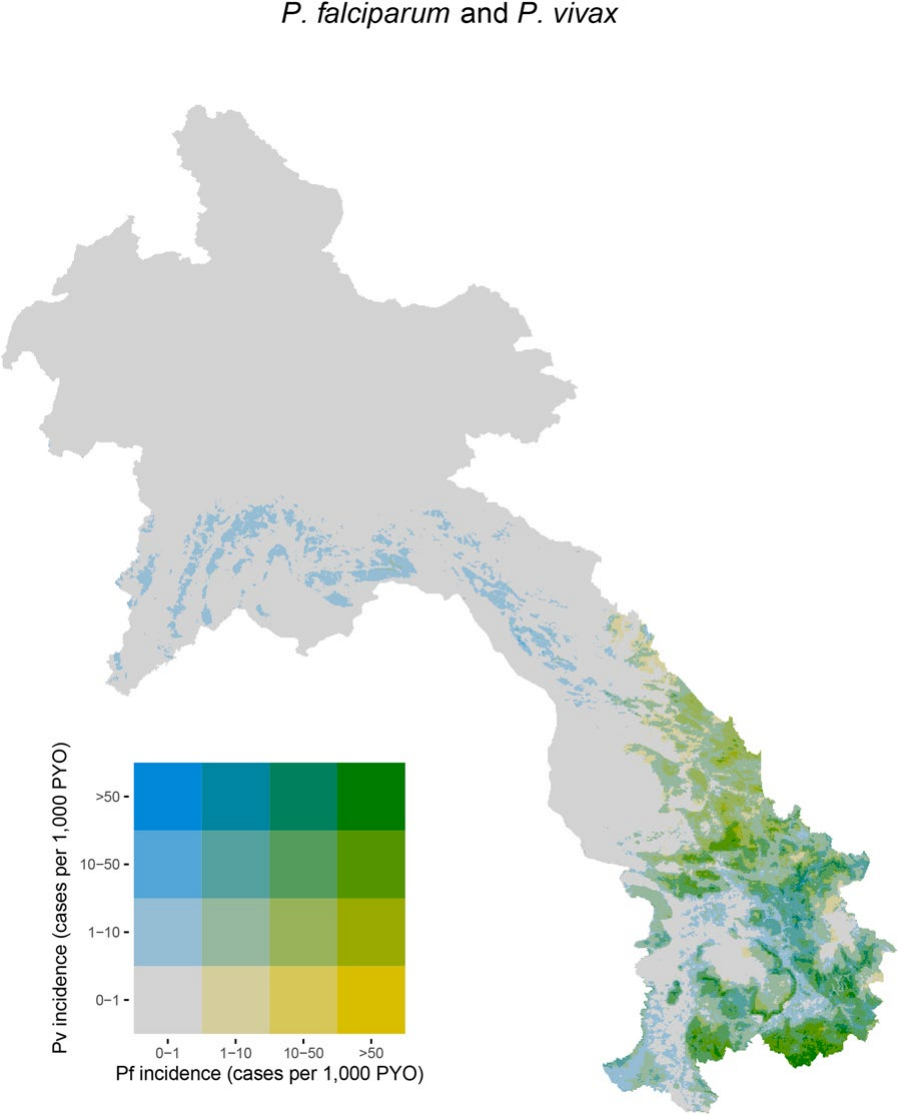
Fine-scale bivariate map of the predicted posterior median of *P. falciparum* and *P. vivax* incidence per 1000 PYO at 1 km $\times$ 1 km across Laos for 2019 to 2021. *P. falciparum* is more spatially spread across the southern provinces compared to *P. vivax*. A notable distinction arises as *P. vivax* exhibits higher prevalence in the central and some northern provinces of the country

The Bayesian geospatial model was applied to species-specific routine surveillance data spanning from 2019 to 2021. As a result, fine-scale incidence maps of the predicted posterior median of *P. falciparum* and *P. vivax* incidence per 1000 PYO were generated at 1 km $\times$ 1 km across Laos (see Figure 5 in SI). The detailed, fine-scale bivariate map (1 km $\times$ 1 km) depicting the incidence of *P. falciparum* and *P. vivax* in Laos from the species-specific incidence model is presented in Fig. 7. The results revealed that *P. falciparum* demonstrates broader spatial distribution within the southern provinces, which include Sekong, Savannakhet, Salavan, Champasak, and Attapeu, in contrast to *P. vivax*. Nevertheless, a significant differentiation emerges as *P. vivax* showcases higher prevalence in the central provinces such as Khammouane and Bolikhamxay, as well as selected northern provinces like Luang Prabang and Xieng Khouang in the country. These findings contribute to the understanding of the distribution and prevalence of the two malaria parasite species, aiding in the design of targeted intervention strategies.

## Discussion

Malaria risk stratification plays a vital role in effective malaria prevention and control planning [35]. The use of temporal and spatial data on malaria incidence in Laos allows for the identification of areas with a high burden of malaria transmission and the detection of changes in risk profiles over time. This information guides the implementation of targeted interventions, resource allocation, and outreach to high-risk populations and has been used to support the 2022 risk stratification planning conducted by CMPE [17] for the Global Fund RAI4 application. The risk maps produced highlight the remarkable effort and successive decline of malaria transmission in the northern regions and persistent transmission that remains predominantly in rural and remote areas, particularly in the southern regions of the country [34]. This work acts as supporting evidence for ongoing opportunities for targeted approaches and accelerator strategies being deployed across southern provinces [36, 37] as well as an opportunity to monitor and maintain gains in the north.

The rising heterogeneity of malaria exposure amidst an overall decline in prevalence, as illustrated in Fig. 2, is a distinctive feature of malaria epidemiology in regions nearing elimination. Similar trends have been observed in previous studies across the GMS, including Cambodia [38], the Yunnan Border [14], Vietnam [39], and Thailand [40]. The spatial heterogeneity of malaria incidence in Laos is influenced by various environmental, social, and economic factors [35]. High risk in urban/rural areas highlights the need for targeted interventions, while hydrology’s role underscores the significance of water bodies. Vegetation’s influence on mosquito habitats suggests environmental management. The interplay of these factors results in the varying risk profiles observed across different regions. To effectively control the disease, targeted interventions in high-risk areas are necessary [15]. These interventions should take into account the local context and focus on addressing the specific risk factors contributing to malaria transmission. For example, across Laos, forested regions are known to be a high risk for transmission and forest-goers remain a high-risk population. Strategies have been put in place to curb the transmission in these populations as part of Laos malaria accelerator strategies towards elimination [41].

The declining incidence of malaria in Laos can be attributed to several factors besides environmental changes [42]. Improved access to effective malaria prevention and control interventions, such as the distribution of insecticide-treated bed nets [16, 43], early diagnosis and treatment, and indoor residual spraying, have played a significant role [44]. Additionally, the establishment of better surveillance and response systems has enhanced the detection and management of malaria cases [45]. Strengthened cross-border collaboration with neighboring countries has also contributed to the overall reduction in malaria transmission in the region [46]. Changes in the risk profile of malaria incidence over time can be influenced by various factors, such as environmental changes, social and economic changes, or the effectiveness of existing control strategies. As malaria transmission decreases in some areas, new hotspots may emerge, necessitating a re-evaluation of operational strategies. Flexibility and adaptability in malaria prevention and control approaches are crucial to respond to changing risk profiles effectively. This includes the maintenance of a strong surveillance system that can identify and monitor malaria cases, including asymptomatic infections and reduced case detection [47].

The reduction in malaria cases in Laos [37] and the GMS [48] is also partly attributed to deforestation, which disrupts habitats of malaria-carrying mosquitoes. As forests are cleared for agriculture, urbanization, and infrastructure, mosquito breeding sites diminish, reducing their population density and malaria transmission [49]. While this unintended consequence aids malaria elimination, it highlights broader environmental and socio-economic impacts. Addressing deforestation necessitates balancing economic development with environmental conservation and public health priorities.

It is important to highlight that the decline in malaria cases in Laos in 2020 and 2021 may be partially linked to the indirect effects of the COVID-19 pandemic. Measures to control COVID-19, such as lockdowns and travel restrictions, likely disrupted the movements of malaria vectors and human populations, thereby reducing malaria transmission. Additionally, public health campaigns related to COVID-19 may have increased awareness about preventive measures against malaria, further contributing to the decline in transmission. However, it is important to note that ongoing malaria control efforts have also played a significant role in reducing malaria transmission in Laos in recent years. According to the WHO, malaria cases in Laos have plummeted from an estimated 462,000 in 1997 to 2305 in 2022 [41]. Moreover, according to the Mekong Malaria Elimination Programme, there were only 96 reported malaria cases in Laos from April to June 2023, marking an 88% decrease compared to the corresponding period in 2022 [50].

The maps provided Fig. 5 not only facilitate the assessment of the prediction accuracy but also provide valuable information about the catchment populations for individual health facilities. By showcasing the observed cases in relation to the predicted cases, the figure serves as a means to gauge the model’s performance and the extent to which it captures the real-world dynamics of malaria cases across different catchment areas. This approach contributes to a comprehensive evaluation of the model’s predictive capabilities and its ability to estimate catchment populations effectively, enhancing the overall reliability of the study’s findings. During stratification, These risk maps were used to identify discrepancies between observed case counts and predicted. Facilities with large deviations were audited more deeply to confirm correct case counts. In the absence of health facility catchment estimations and case information, the model provided predicted estimates to support stratification. The details of the steps taken for Laos stratification are described here [17].

Disaggregated species maps (see SI) and the bivariate map (Fig. 7) presented here hold immense value when it comes to the country’s goal of eliminating *P. falciparum* malaria by 2023 and eliminating *P. vivax* malaria in the 13 Northern provinces by 2025. While *P. falciparum* elimination is the primary objective, *P. vivax* cannot be overlooked due to its unique characteristics and challenges. *Plasmodium vivax* has the ability to form dormant liver stages, leading to relapses and making it more difficult to eliminate completely. Therefore, understanding the specific distribution and transmission patterns of *P. vivax* through disaggregated species maps becomes essential for targeted interventions. By utilizing different strategies for each species, such as targeted treatment regimens, vector control measures [4], and surveillance approaches, Laos can address the distinct challenges posed by *P. falciparum* and *P. vivax*. This comprehensive approach takes into account the evolving dynamics of malaria transmission and ensures that efforts are optimized for both species, ultimately leading to a more effective and sustainable elimination strategy.

Continued investment in malaria prevention and control efforts in Laos is crucial for sustaining the progress made in reducing malaria incidence. This includes ongoing support for surveillance systems, strengthening healthcare infrastructure, capacity building, and community engagement [51]. Sustained efforts are needed to address the remaining challenges, such as the persistence of malaria in high-risk provinces and the potential for the emergence of new hotspots. By maintaining a comprehensive and adaptive approach, Laos can further reduce the malaria burden and progress towards the ultimate goal of malaria elimination.

With regard to the modeling choice, it is notable that both a negative binomial (NegBin) and a zero-inflated Poisson model were evaluated. The NegBin model demonstrated superior performance in terms of goodness-of-fit measures for the data, which exhibited significant overdispersion—a key factor that the NegBin model was particularly adept at handling. Although the NegBin model did not explicitly model the non-independence of cases (underdispersion), it remained effective for the current dataset where overdispersion was the predominant issue [52]. In addition to its empirical suitability, the NegBin model offered computational simplicity and ease of interpretation, which were valuable in complex epidemiological modeling. While the limitations regarding underdispersion were acknowledged, the decision to use the NegBin model was based on its overall utility in capturing the variability observed in the data more accurately than alternative models.

The feasibility of employing a Bayesian spatio-temporal model for the observed dataset was examined. However, it was observed that the temporal component in the resulting annual maps tended to dominate the inter-annual variation in malaria transmission. These oversmoothed outcomes could stem from several factors, including inappropriate temporal aggregation, an inadequate temporal correlation structure, or suboptimal smoothing parameter selection [53]. When data are aggregated over extensive spatial or temporal units, fine-scale variability can be lost, leading to patterns that appear oversmoothed. Coarse-resolution data aggregation has the potential to conceal local variations and create an overall pattern that appears smoother. For the temporal effect, an autoregressive model of first-order (AR1) was employed. Nevertheless, alternative choices such as the Gaussian random walk model of order 1 (RW1) or order 2 (RW2) can be explored in future investigations to address the oversmoothing issue [54]. It is important to note that a highly flexible model with numerous parameters can overfit the data, resulting in smoothed outcomes that may not align with the actual patterns supported by the data. By considering these factors and potential improvements in model specifications, the accuracy and effectiveness of spatio-temporal modelling for malaria transmission can be enhanced.

It is important to acknowledge some limitations of the study. The analysis is based on routine surveillance data, which may be subject to under-reporting or other biases. Additionally, the models rely on assumptions and simplifications that may influence the accuracy of the predictions. Future research could incorporate additional data sources, such as entomological data, socioeconomic factors, and human movement patterns, to further improve the accuracy and understanding of malaria transmission dynamics in Laos. Additional data such as serological surveys can strengthen the accuracy of risk maps as laos proceeds to extremely low numbers [13]. Reactive case detection supplemented by GPS locations of villages can strengthen the accuracy in catchment estimations and provide deeper insight into the dynamic changes that occur year on year.

In conclusion, the study provides valuable insights into the spatial patterns, trends, and species-specific variations in malaria incidence in Laos. The reduction in malaria incidence, particularly in high-risk areas, demonstrates the effectiveness of malaria prevention and control interventions. However, challenges remain, and targeted interventions adapted to the changing risk profiles are necessary. Malaria risk stratification and a flexible operational approach are crucial for sustaining progress and achieving malaria elimination in Laos. Continued investment and collaboration at local, regional, and international levels are essential to overcome these challenges and further advance malaria control efforts in the country.

### Supplementary Information

Supplementary Material 1.

## Data Availability

Data available upon reasonable request to the Center of Malariology, Parasitology and Entomology, Vientiane, Lao PDR.
