# Supplementary material for: Fine-scale maps of malaria incidence to inform risk stratification in Laos
Source: Malar J. 2024 Jun 25;23:196. doi: 10.1186/s12936-024-05007-9 (PMC11202256; doi:10.1186/s12936-024-05007-9)
Supplement: Supplementary file 1 — Supplementary Material 1. [file 12936_2024_5007_MOESM1_ESM.pdf]

Fine-scale maps of malaria incidence to inform risk stratification in Laos – Supplementary Materials

Within this supplementary material, we provide the reader with comprehensive model validation and goodness-of-fit results in tabular and graphical form, as well as supplementary tables and figures. These resources serve to facilitate the evaluation of the Bayesian geospatial models' quality.

Additional tables

The outcomes of cross-validation analyses for various model specifications and two distinct parasite models are displayed in Table S1 and Table S2, respectively. Among the different models tested, the one incorporating both covariates and spatial random effects demonstrated the lowest negative log-likelihood. As a result, this particular model was employed throughout the entire study. Additionally, when comparing the cross-validation analyses for two distinct parasite models, it was observed that fitting *P. falciparum* and *P. vivax* separately yielded superior results compared to a joint modelling approach for both parasites.

The posterior mean and corresponding 95% credible intervals for all model parameters are provided in Table S3. Additionally, Table S4 presents a tabulation of the root mean square error (RMSE) and mean absolute error (MAE) computed between the observed and predicted malaria cases at each health facility across Laos during the period spanning 2017 to 2021.

Table S1 Results of cross-validation analyses for different model specifications.

Model specification	Negative log-likelihood
Model with both covariates and spatial random effects (Model A)	1534.58
Model with covariates only (Model B)	1640.59
Model with spatial random effects only (Model C)	1558.27

Model A exhibited the smallest negative log-likelihood among all the models tested, and consequently, it was selected for use throughout the study.

Table S2 Results of cross-validation analyses for two different parasite models.

	Species disaggregation	Negative log-likelihood
Model A1	Joint modelling of *P. falciparum* and *P. vivax*	2233.31
Model A2	A model for *P. falciparum* + A model for *P. vivax*	893.40 + 1108.99 = 2002.39

Based on the specifications of Model A mentioned in Table 1, we evaluated the performance of two additional models. The results indicate that Model A2 outperforms Model A1, as it exhibits a smaller negative log-likelihood.

Table S3 Posterior mean and 95% credible intervals for the covariates of the geospatial model from 2017 to 2021.

	2017		2018		2019		2020		2021	
Covariates	Mean	95% CI	Mean	95% CI	Mean	95% CI	Mean	95% CI	Mean	95% CI
Access to cities	0.98	(0.38, 1.87)	1.18	(0.49, 1.87)	1.06	(0.52, 1.6)	0.64	(0.11, 1.16)	1.05	(0.48, 1.62)
AI	0.75	(0.2, 1.43)	0.71	(-0.01, 1.43)	-0.15	(-0.7, 0.39)	-0.42	(-0.78, -0.07)	-0.36	(-0.78, 0.06)
Distance to water	0.43	(-0.11, 1.29)	0.57	(-0.15, 1.29)	-0.09	(-0.85, 0.67)	-0.06	(-0.97, 0.85)	0.01	(-0.87, 0.88)
Elevation	-0.10	(-1.44, 1.7)	0.12	(-1.47, 1.7)	0.33	(-1.05, 1.72)	0.07	(-1.28, 1.42)	0.21	(-1.24, 1.67)
Night-time lights	-0.04	(-0.3, 0.29)	-0.08	(-0.44, 0.29)	0.36	(0.05, 0.67)	0.12	(-0.23, 0.48)	0.15	(-0.17, 0.47)
PET	1.15	(0.31, 2.49)	1.40	(0.31, 2.49)	0.57	(-0.44, 1.59)	0.49	(-0.43, 1.41)	0.62	(-0.39, 1.63)
Population density	0.05	(-0.27, 0.52)	0.15	(-0.21, 0.52)	-0.10	(-0.5, 0.29)	0.04	(-0.36, 0.43)	0.31	(-0.11, 0.73)
Slope	-0.03	(-0.48, 0.55)	-0.02	(-0.59, 0.55)	0.44	(-0.06, 0.93)	-0.01	(-0.52, 0.5)	-0.21	(-0.67, 0.25)
TSI	-2.21	(-3.29, -0.26)	-1.38	(-2.51, -0.26)	1.04	(0, 2.07)	0.90	(-0.14, 1.94)	1.18	(0.1, 2.27)
Tree fraction	0.25	(-0.97, 2.04)	0.51	(-1.02, 2.04)	-0.90	(-2.42, 0.63)	0.16	(-1.29, 1.61)	0.11	(-1.46, 1.69)
Rainfall	-0.50	(-1.16, 0.04)	-0.62	(-1.28, 0.04)	-0.01	(-0.65, 0.64)	0.69	(0.24, 1.14)	0.43	(-0.05, 0.92)
EVI	1.14	(0.63, 1.69)	1.08	(0.46, 1.69)	0.99	(0.4, 1.57)	0.80	(0.22, 1.38)	1.09	(0.45, 1.72)
LST Day	-0.24	(-1.02, 0.65)	-0.32	(-1.29, 0.65)	-1.39	(-2.12, -0.67)	-1.16	(-1.88, -0.44)	-1.40	(-2.17, -0.63)
LST Night	2.10	(1.16, 2.59)	1.41	(0.22, 2.59)	0.41	(-0.65, 1.46)	0.12	(-0.92, 1.17)	0.09	(-1.03, 1.21)
TCB	-1.91	(-2.52, -0.7)	-1.60	(-2.49, -0.7)	-0.62	(-1.44, 0.2)	-0.80	(-1.64, 0.04)	-1.01	(-1.97, -0.05)
TCW	-1.26	(-1.8, -0.43)	-1.24	(-2.05, -0.43)	-0.97	(-1.77, -0.17)	-0.81	(-1.58, -0.03)	-0.98	(-1.86, -0.1)

Table S4 Root mean square error (RMSE) and mean absolute error (MAE) between observed and predicted malaria cases from 2017 to 2021.

	2017	2018	2019	2020	2021
RMSE	13.65	14.85	14.91	10.36	11.34
MAE	3.53	3.62	3.75	2.43	2.63

Additional figures

Figure S1 depicts the breakdown of observed malaria cases by *P. falciparum* and *P. vivax* from 2019 to 2021 across health facilities in Laos. Additionally, Figure S2 illustrates the continuous treatment-seeking propensity, estimated using a distance-decay-based model to any health facility. Figure S3 exhibits scatter plots of the observed versus predicted malaria cases derived from the Bayesian geospatial model from 2017 to 2021. Figure S4 shows a scatter plot of the number of malaria cases against the ranked health facilities based on observed cases. The maps in Figure S5 present fine-scale incidence maps of the predicted posterior median of *P. falciparum* and *P. vivax* incidence per 1,000 PYO at 1km × 1km across Laos from 2019 to 2021.

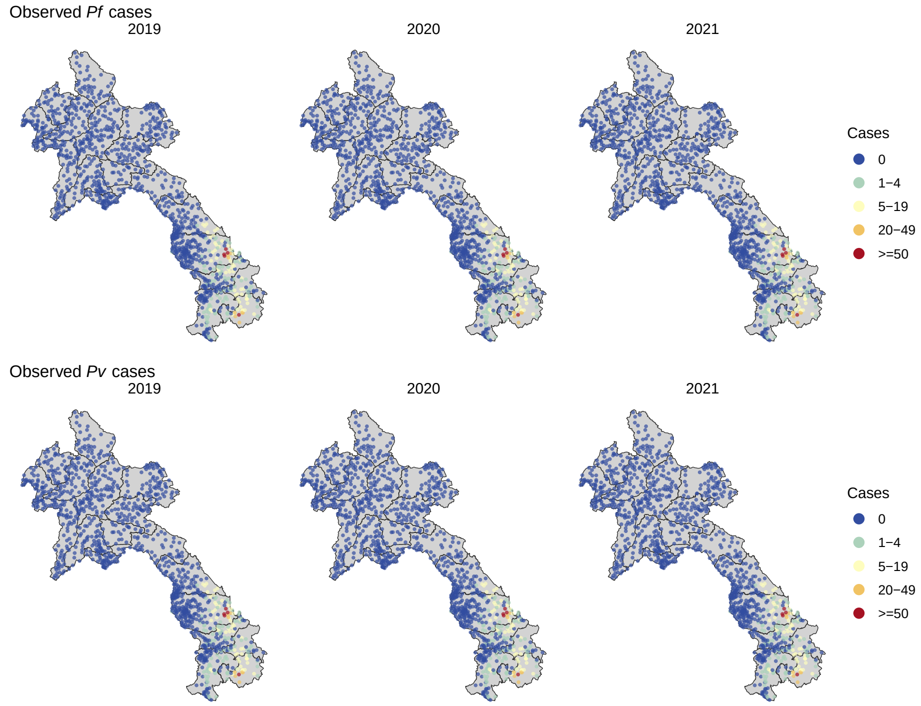

Figure S1 Observed malaria cases disaggregated into *P. falciparum* and *P. vivax* from 2019 to 2021 across health facilities in Laos.

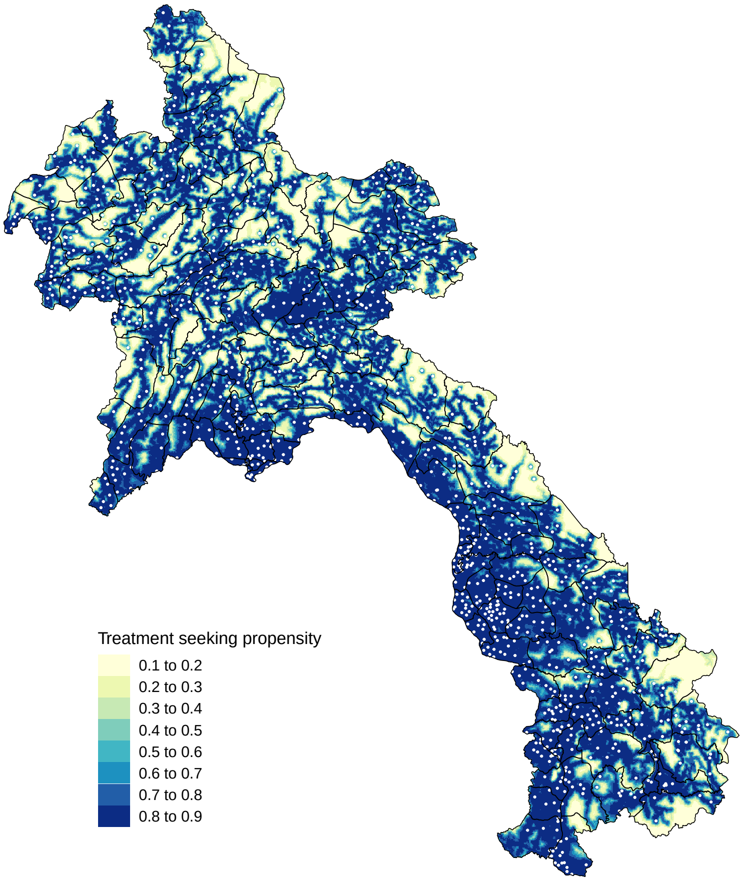

Figure S2 Treatment seeking propensity: the white dots denote health facilities. The map was produced using the health facilities accessibility surface and informed with survey values from the Lao PDR Social Indicator Survey II 2017.

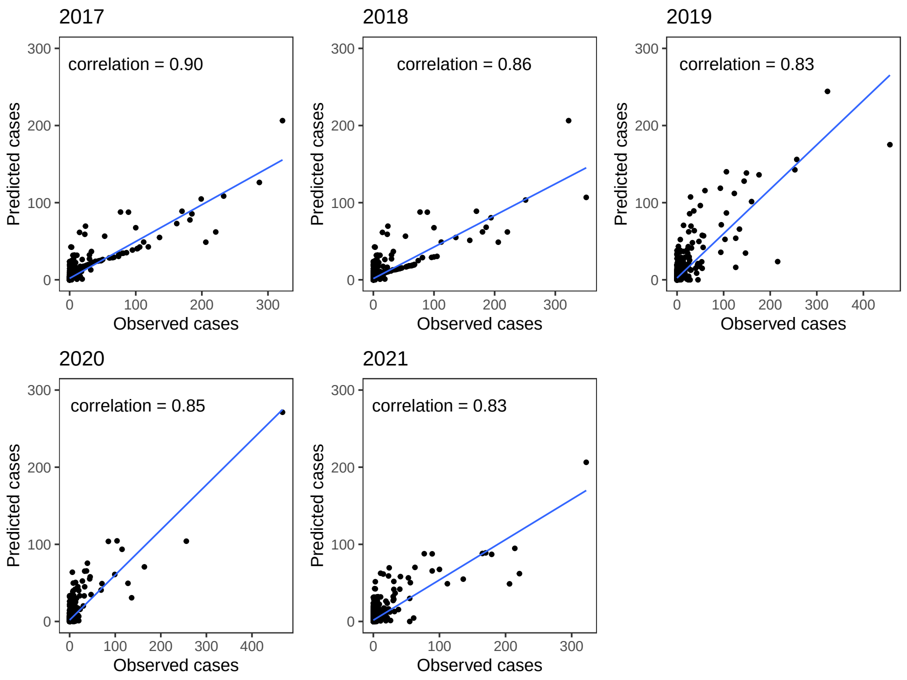

Figure S3 Scatter plot of observed versus predicted malaria cases derived from our Bayesian geospatial model from 2017 to 2021. Correlation values above 0.80 for each year indicate good fits of the modeling approach.

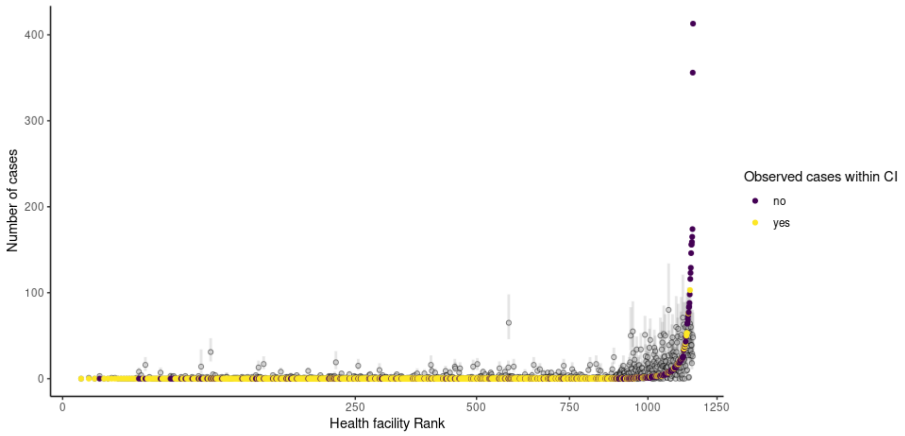

Figure S4 A scatter plot illustrating the relationship between the number of malaria cases and the ranking of health facilities based on observed cases. The grey points represent the predicted cases, accompanied by a credible interval (CI) bound. On the plot, colored dots indicate observed cases per health facility: yellow dots signify observed values within the CI, while purple dots fall outside the CI.

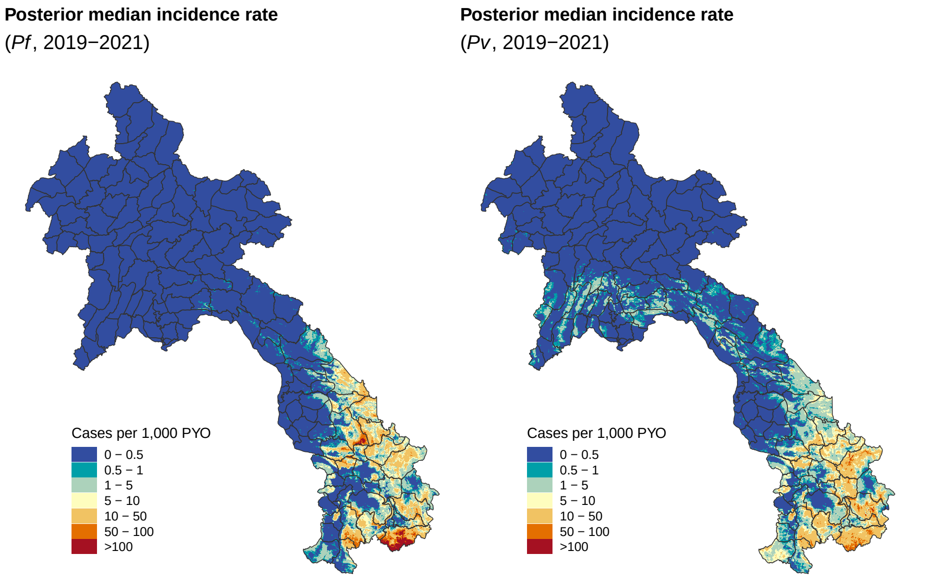

Figure S5 Fine-scale incidence maps of the predicted posterior median of *P. falciparum* (left panel) and *P. vivax* (right panel) incidence per 1,000 PYO at 1km $\times$ 1km across Laos from 2019 to 2021.
